# Soluble HIV-1 Envelope Immunogens Derived from an Elite Neutralizer Elicit Cross-Reactive V1V2 Antibodies and Low Potency Neutralizing Antibodies

**DOI:** 10.1371/journal.pone.0086905

**Published:** 2014-01-23

**Authors:** Sara Carbonetti, Brian G. Oliver, Jolene Glenn, Leonidas Stamatatos, D. Noah Sather

**Affiliations:** 1 Seattle BioMed, Seattle, Washington, United States of America; 2 Department of Global Health, University of Washington, Seattle, Washington, United States of America; Simon Fraser University, Canada

## Abstract

We evaluated four gp140 Envelope protein vaccine immunogens that were derived from an elite neutralizer, subject VC10042, whose plasma was able to potently neutralize a wide array of genetically distinct HIV-1 isolates. We sought to determine whether soluble Envelope proteins derived from the viruses circulating in VC10042 could be used as immunogens to elicit similar neutralizing antibody responses by vaccination. Each gp140 was tested in its trimeric and monomeric forms, and we evaluated two gp140 trimer vaccine regimens in which adjuvant was supplied at all four immunizations or at only the first two immunizations. Interestingly, all four Envelope immunogens elicited high titers of cross-reactive antibodies that recognize the variable regions V1V2 and are potentially similar to antibodies linked with a reduced risk of HIV-1 acquisition in the RV144 vaccine trial. Two of the four immunogens elicited neutralizing antibody responses that neutralized a wide array of HIV-1 isolates from across genetic clades, but those responses were of very low potency. There were no significant differences in the responses elicited by trimers or monomers, nor was there a significant difference between the two adjuvant regimens. Our study identified two promising Envelope immunogens that elicited anti-V1V2 antibodies and broad, but low potency, neutralizing antibody responses.

## Introduction

The HIV-1 epidemic remains a significant global health priority, with 2.3 million new HIV-1 infections and 1.6 million AIDS-related deaths each year (UNAIDS global report, 2013). Despite advances in increasing access to anti-retroviral therapies and the development of microbicides, a universally effective anti-HIV-1 vaccine remains the best hope of defeating the pandemic [1]. Recent findings from the RV144 vaccine trial indicated that protection from infection can be achieved by vaccination [2], and that antibodies were a major contributor to this protection [3]. Antibodies that target an epitope within the variable regions V1 and V2 of the HIV-1 Envelope protein (Env) have been linked to a reduced risk of acquisition in this trial [3]. Additionally, a sieving effect at two positions in the V1V2 region was recently reported in breakthrough infections in the trial, providing evidence of antibody selection pressure within the V1V2 region [4]. These findings build on a long history of experimental studies in non-human primates indicating that, if present prior to infection with HIV-1, anti-HIV-1 neutralizing antibodies can effectively block infection with the virus [5]–[8].

A protective antibody response against HIV-1 will likely need to include antibodies that neutralize a wide array of distinct genetic viral isolates [9], [10]. The sole target of neutralizing antibodies, Env, is a problematic vaccine target due to extreme genetic variability and a high degree of glycosylation [11]. Some degree of neutralizing breadth has been achieved by vaccination, but current generations of Env subunit vaccines have failed to elicit the exceptionally broad and potent anti-HIV-1 neutralizing antibody responses likely necessary to achieve sterilizing protection. However, broadly neutralizing antibodies (bNAbs) have been isolated from HIV-1 infected human subjects [12]–[21]. These antibodies neutralize a wide array of isolates from multiple genetic clades and serve as a model for the types of antibodies that need to be elicited by vaccination.

These antibodies target several different well-defined conserved epitopes on the HIV-1 Env and have several common features that help inform vaccine design. The majority of these anti-HIV-1 bNAbs have undergone extensive somatic hypermutation and may diverge from the germline-encoded B cell receptor (BCR) by as much as 46% [16], [22]–[24]. Many of these bNAbs have developed long third complementarity determining regions (CDRH3s) which, in several well-characterized cases, allows them to penetrate deep into the conserved regions of Env [14], [15], [23], [25]–[27]. Some of these bNAbs are also thought to be auto-reactive [28], [29]. Therefore, it is likely that eliciting similar antibodies by vaccination will require immunogens and vaccination regimens that are able to drive antibody responses to conserved epitopes and drive extensive antibody maturation.

Recent studies indicate that the development of bNAb responses occurs within the first three years of infection, although it is not clear how such potent antibodies develop during the course of natural infection [30], [31]. It is possible that the phenotype of the circulating viral Envs in such subjects contributes to the development of neutralizing breadth. Potentially, the Env species circulating in these individuals are uniquely suited to stimulate the development of bNAbs and may prove to be exceptional vaccine candidates, as was recently suggested [32]. Indeed, a previous study by Zhang and colleagues reported that such an Env immunogen, gp140_R2_, elicited extensively cross-reactive antibodies by vaccination [33]. To further explore the possibility that Envs isolated from donors with broadly neutralizing activity have unique immunogenic properties, we evaluated gp140 Env immunogens derived from the circulating HIV-1 isolates in an individual who developed especially broad and potent anti-HIV-1 neutralizing antibodies that target the CD4-BS. To this end, we produced four gp140 Env immunogens derived from circulating isolates in subject VC10042, characterized their antigenic profiles and evaluated their ability to elicit anti-HIV-1 neutralizing antibodies.

## Materials and Methods

### Donor Subject

Subject VC10042 is an HIV+ African American male who acquired HIV-1 clade B in 1984 and is part of the Vanderbilt cohort, as previously reported [34]. Plasma samples were obtained for multiple time points between 19 and 22.5 years post sero-conversion. Analysis of the anti-HIV-1 serum neutralizing activity has been previously reported [34]–[36]. At the time of sample collection, VC10042 was co-infected with both Hepatitis B and Hepatitis C, both of which were acquired at an unknown time. During the period of observation, subject VC10042 maintained steady CD4+ T cell counts, was without anti-retroviral therapy, and had no AIDS-defining illness.

### Design and Production of Envelope gp140 Trimeric Immunogens Derived from VC10042

The cloning of *envelope* sequences from subject VC10042 has been described previously [35]. Four clones, VC10042.05, VC10042.05.RM, VC10042.08, and VC10042.e1a, were selected for production as soluble gp140 trimeric immunogens. The *env* sequences were modified to express as gp140 soluble trimers by introducing a stop codon at the C-terminus of the membrane proximal external region (position 683-HXB2 numbering) and removing the primary and secondary cleavage sites, as previously described [37]–[39]. Additionally, the native leader sequence was replaced with the tissue plasminogen activator (tPA) leader sequence to increase expression levels [40]. The sequences of the gp140s are deposited in GenBank with sequential accession numbers KF753691–KF753694.

The modified, codon optimized sequences were cloned into the pTT3 vector [41] for transient expression in mammalian cell culture as previously described [40], [42]. Briefly, gp140 immunogens were produced in HEK293F mammalian cells by high density transfection (12 µg/ml DNA added to 20 M cells/ml) using PEI Max (Polysciences, Warrington, PA, USA) as a transfection reagent. After three hours, the culture was diluted to 1 M cells/ml and allowed to grow for six days. The culture supernatants were harvested, clarified by centrifugation, concentrated by tangential flow filtration, and buffer exchanged into 20 mM Tris, pH 7.4, 100 mM NaCl. The gp140 proteins in the supernatant were bound to a lectin chromatography column (derived from *Galanthis nivalis*), which binds the sugar moieties on the surface of the protein, and were eluted in 20 mM Tris, pH 7.4, 100 mM NaCl, 1 M methyl-α-D-mannopyranoside. Following lectin affinity purification, the monomer and trimer fractions were separated by size exclusion chromatography on a Superdex 200 26/60 HiLoad gel filtration column (GE Healthcare, Pittsburg, PA).

### Purification of MLV gp70-V1V2 Scaffolds

Murine leukemia virus (MLV) gp70-V1V2 scaffolds were constructed by joining the HIV-1 Env contiguous V1V2 region amino acid sequence to the C terminus of the first 263 amino acids of the MLV gp70, as previously described [43]. In addition, we produced a variant containing only the first 263 amino acids of MLV gp70. The endogenous leader peptide was replaced with the tPA leader sequence to increase expression levels and the entire sequence was cloned into the pTT3 vector for use in transient transfection in HEK293F suspension cell culture, as described above. The transfection, expression, and purification methods for these scaffolds are exactly identical to the protocols that were utilized for purifying the gp140 Envs as described above. The monomeric form of each gp70-V1V2 was used in all of the analytical assays in this study. The V1V2 sequences were derived from the consensus clade A, B, and C *env* consensus sequences from the Los Alamos sequence database (http://www.hiv.lanl.gov/). The amino acid sequence of each MLV gp70-V1V2 protein is shown in Figure S1 in File S1.

### Ethics Statement

All of the animal studies described in this manuscript were carried out in accordance with the recommendations described in the Guide for the Care and Use of Laboratory Animals (Eighth Edition, National Academies Press, 2011, Washington DC, USA). Immunizations were carried out in New Zealand white rabbits (*Oryctolagus cuniculus*) at the Pocono Rabbit Farm and Laboratory, Inc. (Canadensis, PA, USA) (OLAW Assurance # A3886-01, AAALAC accreditation). This study protocol was reviewed, approved, and supervised by the Institutional Animal Care and Use Committee at Seattle Biomedical Research Institute (internal protocol entitled, “NS-ABP”). All procedures involving harvesting samples from immunized animals were carried out under anesthesia.

### Immunizations

This study utilized a total of 36 animals in 12 immunization groups (three animals per group). One animal, 28267, died during the study of causes unrelated to the study. Each animal received four monthly immunizations of a protein/adjuvant mix (100 µg/100 µg) or protein alone (100 µg) and serum samples were collectedtwo weeks after each immunization. Each immunization consisted of two intra-muscular injections in the hind legs, with each injection containing 50 µg protein immunogen and 50 µg QS-21 in 100 µl total volume (protein+adjuvant). QS-21, a saponin derived from the *Quillaja saponaria* tree, was used as an adjuvant (Antigenics, Lexington, MA, USA) [44]–[47]. Two immunization regimens were evaluated that differed in the number of immunizations that contained QS-21 adjuvant. In the first, animals received adjuvant at all four immunizations (designated “4A”). In the second regimen, animals received adjuvant only at the first and second immunizations (designated “2A”), while the third and fourth immunizations did not contain adjuvant (see Figures S2 and S3 in File S1). Trimers were tested in both the 2A and 4A protocols, and monomers were tested only in the 2A protocol.

### ELISAs

Enzyme linked immunosorbent assays (ELISAs) were performed to evaluate both the antigenic characteristics of the Env immunogens and to evaluate serum immune responses in immunized animals. Antigens were adsorbed onto the wells of 96-well Immulon 2HB plates (Thermo, Waltham, MA, USA) overnight at room temperature (RT) in 100 mM NaHCO_3_ pH 9.4 for protein antigens and 200 mM NaHCO_3_, pH 9.4 for peptide antigens. After washing, the plates were blocked in phosphate buffered saline (PBS) containing 10% non-fat milk (NFM) and 0.3% Tween-20. Antibodies or immune serum were diluted in PBS, 10% NFM, 0.03% Tween-20 for the primary incubation of 60 minutes at 37°C. After washing, HRP-labeled secondary antibodies, diluted in PBS, 10% NFM, 0.03% Tween-20, were added for one hour at 37°C. After the final round of washes, the reactions were developed in 1-Step Ultra TMB-ELISA (Thermo Scientific, Waltham, MA, USA) for 4 minutes at room temperature, stopped in equal volumes of 1 N H_2_SO_4_, and absorbance at 450 nm was determined on a SpectraMax M2 microplate reader (Molecular Devices, Sunnyvale, CA, USA). Endpoint titers were defined as the highest dilution (or MAb concentration) producing absorbance readings of 3-fold above background. MAb VRC01 was kindly provided by J. Mascola (VRC, NIH) and MAbs 4E10, 2F5, 2G12, and b12 were purchased from Polymun Scientific (Vienna, Austria). PG9 and PG16 were provided by Theraclone (Seattle, WA, USA). CD4-IgG2 was purchased from Progenics Pharmaceuticals (Tarrytown, NY, USA). For serum ELISAs, endpoint titers were compared among the experimental groups by One- and Two-way ANOVA and by Mann-Whitney U test with a significance cutoff of 0.05.

### Neutralization Assays

Neutralization assays were performed using the TZM-bl cell-based pseudovirus assay, as previously described [48]. Immune serum were tested for neutralizing activity against Env clones from clades A [49], B [50] and C [51]. Pseudovirus bearing the MLV Env was used in all assays to determine the level of non-specific neutralizing activity. Briefly, serum samples were diluted 1∶5 and mixed with equal volumes of pseudovirus, for a final dilution of 1∶10, for ninety minutes at 37°C. The virus/serum mixture was added to TZM-bl cells that were plated at a density of 3×10^3^ cells per well in a 96 well plate 24 hours prior to inoculation. 72 hours later the cell supernatants were removed and 100 µl of SteadyLite Plus (Perkin Elmer, Waltham, MA, USA) was added to the cells of each well. The cell-associated luciferase activity (luminescence) for each well was determined on a Fluoroscan Luminometer (Thermo Scientific, Waltham, MA, USA). For immune serum, the neutralization value reported here is the percent neutralization at a single concentration, 1∶10, and is the average of triplicate assays. For assays involving human plasma, we report the reciprocal IC50, which is the dilution at which neutralization was reduced to 50%. Pseudo-typed MLV Envelope was used to test for non-specific neutralizing activity in the serum, and if present, we subtracted three times the MLV background for all neutralization values for that given serum. The neutralizing responses were compared by Two-way ANOVA and by Kruskal-Wallis test (non-parametric One-way ANOVA) with significance cutoffs of p = 0.05.

### Luminex Assays

Peptides corresponding to the variable regions V1–V5 of the consensus clade B Env (see Table S1 in File S1 for complete peptide sequences) were synthesized by Genscript (Piscataway, NJ, USA) at >85% purity. 12 µg peptide was coupled to 1 M Bio-Plex COOH beads (BioRad, Hercules, CA, USA) by primary amine coupling utilizing sulfo-NHS attachment chemistry, according to the manufacturer’s protocol. Immune and pre-bleed serum samples were diluted 1∶10 in PBS and mixed with peptide-coupled beads (2000 beads per peptide) on a filter bottom plate (Millipore, Billerica, MA, USA) and incubated at RT for one hour on a shaker at 600 RPM. After washing, phycoerytherin-labeled secondary antibody diluted 1∶500 in PBS, 0.02% Tween-20 was added for 1 hour with shaking. Goat α-rabbit-PE secondary antibody was used detetct rabbit serum antibodies, and goat α-human-PE secondary antibody was used for human plasma antibodies (Southern Biotech, Birmingham, AL, USA). After 5 final washes of 5 minutes each in PBS, 0.02% Tween-20, the beads were analyzed for binding on a Luminex 200 (Invitrogen, Carlsbad, CA, USA), and the net median florescence intensity (net MFI) is reported, minus the net MFI of matched pre-bleed samples.

## Results

### gp140 Env Immunogens Derived from Subject 10042

We recently isolated envelope sequences from VC10042, an HIV-1 infected elite neutralizer whose serum antibodies are able to potently neutralize a wide array of HIV-1 isolates from clades A, B, and C [35]. For this study, we selected four *env* clones to produce as soluble gp140 immunogens (Table 1). Env immunogens VC10042.05 and VC10042.05.RM were derived from the same *env* clone and differ only by a single amino acid change, R373M (HXB2 numbering). This single amino acid change was found to make the neutralization-resistant clone VC10042.05 sensitive to the autologous anti-CD4-BS NAbs circulating in VC10042, and was found to modulate sensitivity to anti-CD4-BS bNAbs VRC01 and NIH45-46^G54W^ [16], [19], [35], [52]. Thus, the parental *env* clone for VC10042.05 was resistant to most anti-CD4-BS bNAbs, whereas the parental *env* clone for VC10042.05.RM exhibits an increased sensitivity to anti-CD4-BS bNAbs. These two closely related gp140 sequences derived from VC10042.05 are 663 amino acids in length, and contain 35 N-linked glycosylation sites (NLGS) (Table 1).

**Table 1:** Sequence characteristics of gp140 Env immunogens derived from VC10042.

Env gp140	LENGTH (A.A.)^1^	NLGS^2^ (number)	V1V2 LENGTH (A.A.)	V3 LENGTH(A.A)	V4 LENGTH(A.A.)	V5 LENGTH(A.A)
10042.05	663	35	80	35	32	9
10042.05.RM	663	35	80	35	32	9
10042.08	667	32	84	35	33	9
10042.e1a	647	23	70	35	30	9

1 A.A. = amino acid residues.

2 NLGS = N-linked glycosylation site.

Clones VC10042.08 and VC10042.e1a exhibited drastically different neutralization phenotypes than VC10042.05. Both clones were sensitive to anti-CD4-BS bNAbs b12, VRC01, and NIH45-46^G54W^ [35], [52]. Clone VC10042.08 was extremely sensitive to the autologous plasma, whereas VC10042.e1a was only minimally neutralized by the autologous plasma [35]. In contrast to the other clones, VC10042.e1a was very sensitive to bNAbs that target an epitope that overlaps with the CD4-BS, termed the “core” epitope [13]. The gp140 sequence derived from VC10042.08 is 667 amino acids in length, and contains 32 NLGS. The gp140 sequence derived from VC10042.e1a is 647 amino acids and has 23 NLGS. Thus, the four gp140 Env immunogens chosen for this study represent a range of neutralization phenotypes and sensitivities, amino acid lengths, and number and patterns of N-linked glycosylation motifs.

### Antigenic Properties of gp140 Trimeric Envs Derived from VC10042

The trimeric and monomeric gp140 Env proteins derived from VC10042 were tested for their ability to bind known broadly neutralizing monoclonal antibodies by ELISA. All Env immunogens, both in trimeric and monomeric forms, bound the anti-V3 MAb 447-52D [21], and all but VC10042.e1a bound to MAb 2G12, a MAb that targets a cluster of high mannose residues on gp120 (data summarized in Table 2 and shown in Figures S4–S7 in File S1) [12]. MPER-directed MAb 4E10 and to a lesser extent, 2F5, bound to Envs VC10042.e1a and VC10042.08, but did not bind VC10042.05 and VC10042.05.RM.

**Table 2:** Antigenic characteristics of VC10042-derived Envs.

gp140 Env	VRC01	CD4-IgG2			MPER	2G12	447-52D	PG9
10042.05	−1		+++^2^	−		++	+++	−
10042.05(m)^3^	+		+++	−		++	+++	+
10042.05.RM	+		+++	−		++	+++	−
10042.05.RM (m)	++		+++	−		++	+++	+
10042.e1a	+++		+++	++		−	+++	+
10042.e1a (m)	++		+++	++		−	+++	+
10042.08	+		+++	+		++	+++	−
10042.08 (m)	+		+++	+		++	+++	+

1 (−) = no binding.

2 (+) = relative strength of binding is denoted by an increasing number of + symbols.

3 (m) = monomeric form.

The four gp140s exhibited a range of binding characteristics to antibodies that target the CD4-BS. MAb b12 bound only to VC10042.e1a, but not the other three gp140s. MAb VRC01 bound to all gp140s with the exception of VC10042.05, although weak binding was detected to VC10042.05 monomer. CD4-IgG2, a chimeric antibody-CD4 receptor molecule [53], bound robustly to all four gp140s, indicating that the CD4-BS was structurally intact in all four proteins. Interestingly, we observed differing binding characteristics between the monomeric and trimeric forms of each protein. For example, MAb PG9, which recognizes a glyco-peptide epitope in the V2 region, only exhibited weak binding to one of the four trimeric immunogens (VC10042.e1a). In contrast, weak to moderate binding of PG9 was recorded for all four immunogens in their monomeric forms. PG16 did not bind to any of the four immunogens in any form.

The antigenic profile of the VC10042-derived gp140 Env proteins did not always match the neutralization profiles for the parental *env*. For example, while the anti-V3 MAb 447-52D bound to all four Envs, none of the parental viruses were susceptible to neutralization by this MAb [35]. Additionally, the gp140 proteins (with the exception of VC10042.e1a) bound to MAb 2G12, but none of the parental *envs* were sensitive to neutralization by 2G12. MAb b12 neutralized both VC10042.e1a and VC10042.08, but bound only to VC10042.e1a and did not bind VC10042.08 by ELISA. However, in several cases the antigenic profile matched the neutralization profile. For example, 10042.e1a bound VRC01 and the parental *env* was exquisitely sensitive to neutralization by VRC01. Additionally, none of the VC10042-derived gp140s bound to MAb PG16, and, in agreement, none of the parental *envs* were sensitive to that MAb [35]. Thus, the exposure of certain conserved epitopes was not necessarily the same between the native virion-associated trimers and the soluble, recombinant gp140 trimers produced for immunization studies.

### VC10042-derived gp140s are Immunogenic in Rabbits

We tested the immunogenicity of the gp140 Envs in New Zealand White rabbits (*Oryctolagus cuniculus*). Each immunization group consisted of three animals, which received a total of four monthly intramuscular immunizations of 100 micrograms of gp140 Env mixed 1∶1 with 100 µg QS-21 as an adjuvant. Serum samples were collected two weeks after each injection (see Figures S2 and S3 in File S1). We tested two different adjuvant regimens for immunizations with trimeric gp140. In the first groups, animals received adjuvant at only the first and second immunizations (abbreviated as 2A), whereas the third and the fourth immunizations did not contain adjuvant. In the second group, animals received adjuvant with all four immunizations (abbreviated 4A). In addition, the four immunogens described above also were tested as monomers, but only in the 2A regimen for direct comparison against the trimer 2A regimen.

**Figure 1:**
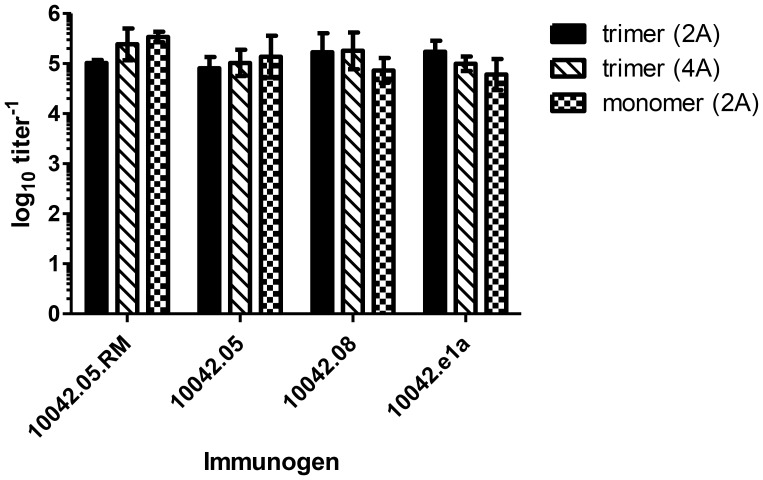
Comparison of vaccine-elicited ELISA titers. The magnitude of antibody binding titers after the fourth immunization was compared among immunization groups by Two-way ANOVA. (2A) = adjuvant given at only the first two immunizations; (4A) adjuvant given at all four immunizations.

We monitored the serum antibody responses for both binding and viral neutralization at several time points during the immunization protocol. All of the four immunogens, both as monomeric and trimeric proteins, elicited high endpoint titers of binding antibodies after the second immunization (Table S2 in File S1), which remained relatively stable after the third and fourth immunizations. The antibody binding titers elicited by both the monomeric and trimeric forms of each immunogen were similar in magnitude (p = 0.882), indicating that the two protein forms elicit similar levels of binding antibodies (Figure 1; Table S2 in File S1). In addition, the binding titers elicited in those animals that received adjuvant at only the first two trimer immunizations (2A) and those that received adjuvant at all four trimer immunizations (4A) were comparable in magnitude (p = 0.561). Overall, neither the immunogen (p = 0.11) nor the vaccine regimen (p = 0.75) had a statistically significant impact on the post fourth immunization Env binding titers (Figure 1). Thus, the continued use of adjuvant over the entire course of the immunization regimen did not offer a significant advantage in increasing the overall magnitude of the vaccine-elicited antigen binding antibody responses. Additionally, these findings indicate that protein immunizations alone after the initial protein plus adjuvant prime/boost were sufficient to stimulate antibody titers similar to those of the groups that received adjuvant at all four immunizations.

### Linear Responses Target the V2 and V3 Regions

**Figure 2:**
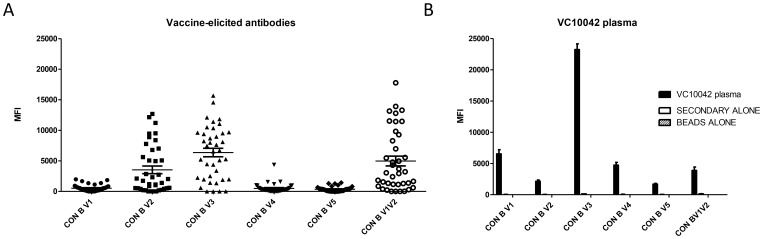
Vaccine-elicited antibody responses to the variable regions of Env. Antibody binding of immune serum (A) to linear peptides corresponding to the variable regions of Env as measured by Luminex assay and compared to the VC10042 donor plasma (B). Each point represents the pre-bleed subtracted net median fluorescence intensity (MFI) from duplicate experiments. The sequences of the peptides used in this assay are listed in Table S2 in File S1.

To assess whether the variable regions V1–V5 were immunogenic in the gp140s, we generated peptides corresponding to the variable regions of the consensus clade B Env sequence and tested them for immune serum reactivity by Luminex binding assays (Figure 2A). In addition to each V region, we also generated a linear peptide corresponding to the V1V2, as these regions are contiguous within the native Env protein (see Table S1 in File S1 for the sequences of the peptides used in this study). In all animals we observed little to no reactivity against peptides corresponding to the regions V1, V4 and V5 of Env. We observed robust binding to the consensus B V3 peptide in all samples, and in most cases we also observed reactivity to the linear V2 peptide. In addition, many of these sera also bound strongly to the linear V1V2 peptide. The binding profiles that we observed are in contrast to those of the original donor plasma from subject VC10042. Although this subject’s plasma bound the V3 peptide to a high degree, we observed little or no reactivity to the V2 or V1V2 peptides (Figure 2B), indicating that the V1V2 was not immunogenic during the course of natural infection in subject VC10042.

**Figure 3:**
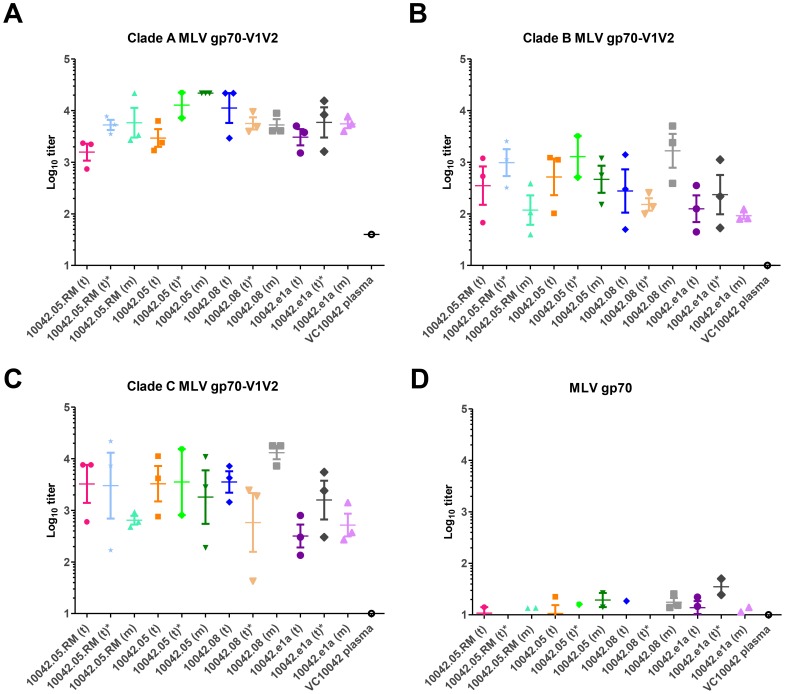
Vaccine-elicited anti-V1V2 antibodies. Anitbody binding was measured by ELISA to gp70-V1V2 constructs derived from clade A (A), B (B) or clade C (C) Envs, and to the MLV gp70 fragment alone (D). Immunogens are differentiated by their monomeric (m) or trimeric (t) forms. Asterisks indicate the use of full adjuvant protocol (4A).

The high degree of reactivity to the linear V2 and V1V2 peptides that we observed by Luminex led us to investigate whether the gp140 immunogens also elicited antibodies that bind the scaffolded HIV-1 V1V2 peptide in the context of the MLV-HIV-1 chimeric gp70-V1V2 protein [43] (Figure 3A–D). This construct is thought to present the HIV-1 V1V2 in a conformation similar to that of the HIV-1 Envelope, but is presented as part of the murine leukemia virus (MLV) envelope protein. Antibodies that bind to this construct have been implicated in the partial efficacy observed in the RV144 Thai vaccine trial and their presence was inversely correlated with the risk of HIV-1 acquisition among vaccinees [3].

All of the gp140 immunogens in this study elicited cross-reactive antibodies that bound to gp70-V1V2 constructs derived from consensus clade A V1V2 (Figure 3A), consensus clade B V1V2 (Figure 3B) or consensus clade C V1V2 (Figure 3C). The four immunogens elicited varying levels of anti-V1V2 antibodies, both as trimer and monomer. The VC10042.05- and VC10042.05.RM-vaccinated groups elicited higher binding titers, whereas VC10042.e1a-vaccinated groups consistently had the lowest binding titers (p = 0.035). Competition neutralization assays, in which the MLVgp70-V1V2 scaffolds were used to compete neutralizing activity in a TZM-bl assay format, indicated that the anti-V1V2 antibodies present in the immune sera do not contribute to the neutralizing activity we observed (see below, data not shown). Interestingly, the VC10042 donor plasma showed little or no reactivity toward the V1V2 scaffolds derived from clades B and C, and only minimal reactivity toward the V1V2 scaffold derived from clade A (Figure 3 A–C). Thus, anti-V1V2 responses in the context of presentation on the MLV gp70 scaffold did not readily occur naturally in VC10042 during the course of infection, similar to our observations with the linear V1V2 responses (Figure 2B). Taken together, these findings imply that the exposure of the V1V2 in the native Envs circulating in VC10042 during natural infection and their cognate soluble Env proteins are significantly different.

### Immunizations with 10042-derived gp140s Elicit Broad, but Low Potency NAb Responses

**Figure 4:**
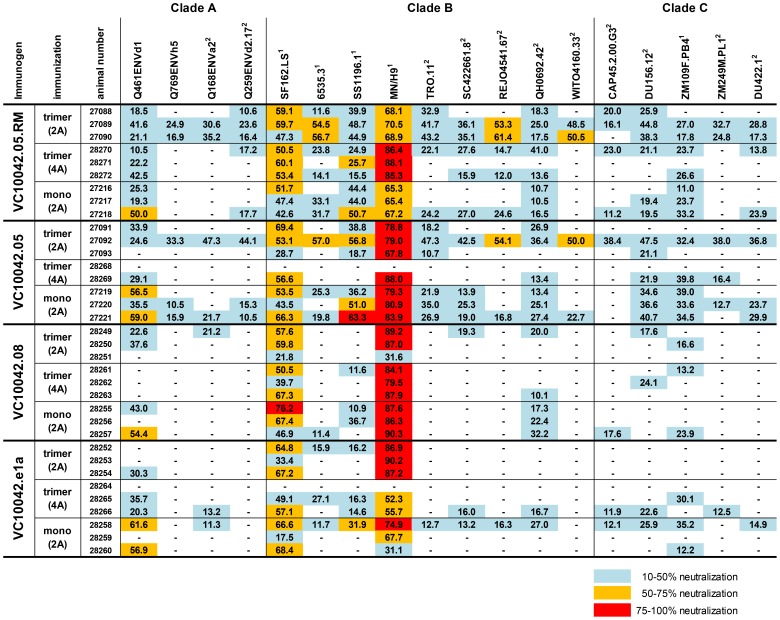
Heterologous neutralizing responses elicited by VC10042-derived Envs. Neutralizing activity of immune serum to 18 clade A, B, and C HIV-1 isolates was measured in the TZM-bl assay. When available, the tier designation is noted in superscript [60]. The number listed is the percent neutralization at a serum dilution of 1∶10, minus three times any MLV activity. In those cases in which greater than 10% neutralization was recorded, the values are heat map color coded. (2A) = adjuvant supplied at only the first two immunizations; (4A) = adjuvant supplied at all four immunizations; trimer = animals immunized with trimer; mono = animals immunized with monomer; (−) = no neutralization was observed.

We analyzed the immune serum for the presence of anti-HIV-1 neutralizing activity using the TZM-bl pseudo-virus assay. Each serum was tested against a panel of viruses consisting of 18 Env clones derived from Clade A (4 Envs), Clade B (9 Envs), and Clade C (5 Envs), and which represent a range of neutralization sensitivities (Tier 1–2), and against MLV Env as a negative control to assess non-specific neutralizing activity. For each immunization group, we recorded neutralizing activity against both tier 1 and tier 2 heterologous HIV-1 isolates, although the potency of neutralization that we observed was very low and in most cases failed to reach 50% (Figure 4). The groups immunized with Envs VC10042.05.RM and VC10042.05, which differ by only a single amino acid at position 373 [35], exhibited neutralizing activity against the largest number of HIV-1 isolates, and in two animals from these groups, 27089 and 27092, we observed some level of neutralizing activity against 100% of the HIV- 1 isolates that were tested (Figure 4). Groups immunized with VC10042.08 and VC10042.e1a exhibited neutralizing activity against far fewer isolates (Figure 4). Thus, immunogens VC10042.05 and VC10042.05.RM elicited antibody responses of greater breadth than VC10042.08 and VC10042.e1a (p = 0.0107, Kruskal-Wallis test).

Of the three variables tested in this study (immunogen, adjuvant, and protein species) only the choice of immunogen (i.e., VC10042.05.RM) had a statistically significant effect on the elicitiation of a more broad neutralizing response (p = 0.0098, Two-way ANOVA). There was no statistically significant difference between the breadth elicited by trimeric gp140s and those elictited by monomeric gp140s (Figure 4), indicating that neither immunogen species elicited a more broad response(p = 0.0591, Kruskal-Wallis test). Additionally, there were no statistically significant differences between the groups that received trimer immunizations with adjuvant at only the first two immunizations (2A) and those that received adjuvant at all four immunizations (4A)(p = 0.111, Kruskal-Wallis test). Thus, in this study, there appeared to be few differences in the neutralizing antibody responses elicited by gp140 trimers and gp140 monomers, nor was there a measurable advantage to providing adjuvant at each immunization.

**Figure 5:**
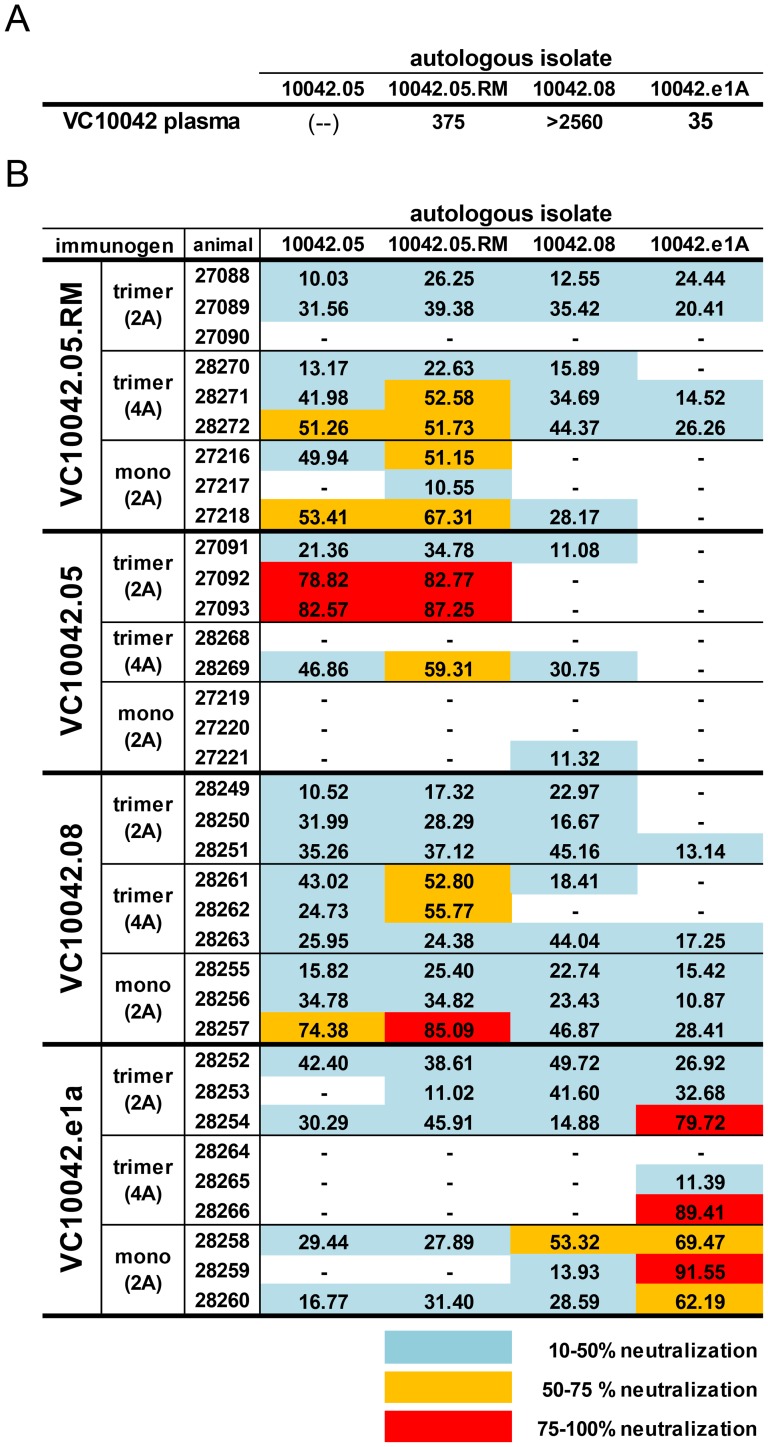
Autologous neutralizing activity elicited by the VC10042-derived Envs. (A) Neutralization of VC10042 viruses by VC10042 plasma. Values reported are the reciprocal IC50 titers, which have been reported previously elsewhere [35], [52]. (B) Neutralization activity of immune serum to VC10042 autologous isolates. The value reported is the percent neutralization at a serum dilution of 1∶10, minus three times any MLV activity. Greater than 10% neutralization was recorded and the values are heat map color-coded. (2A) = adjuvant supplied at only the first two immunizations; (4A) = adjuvant supplied at all four immunizations; trimer = animals immunized with trimer; mono = animals immunized with monomer; (−) = no neutralization was observed.

Immune sera were also tested for their ability to neutralize the autolgous viruses (viruses bearing the same Env from which the immunogens were derived). In general, the potency we observed against the autologuos isolates was far greater than against the heterologous isolates (Figure 5), even though the VC10042 isolates are known to be very resistant to neutralization [35], [52]. Interestingly, several animals neutralized the VC10042.05 autologous isolate (Figure 5B), which is not neutralized by VC10042 plasma (Figure 5A; [35]) and is neutralized only by a single bNAb, NIH45–46^G54W^ [52]. In contrast, only one animal exibited significant neutralizing activity toward VC1002.08, even though this isolate was potently neutralized by VC10042 donor plasma. Thus, the autologous neutralization of the VC10042 plasma did not appear to consistently match the autologous activity elicited by the VC10042-derived Envs. Additionally, the autologous neutralizing activity of the immune sera did not appear to correlate with the degree of heterologous neutralizing activity in these animals, as several animals that neutralized the autologous virus failed to exhibit any degree of heterologous neutralizing activity (animal 27093, for example; Figure 4, Figure 5B). Thus, although several animals exhibited autologous neutralizing activity against highly neutralization resistant VC10042 isolates, it is unclear whether this activity is relevant to the development of heterologous neutralization.

## Discussion

The development of an immunogen and immunization regimens that can elicit broadly neutralizing anti-HIV-1 antibodies remains a top priority. In this study we evaluated four gp140 HIV-1 Envelope immunogens derived from the circulating viruses in an elite neutralizer, subject VC10042, to evaluate whether such Envs possess unique immunogenic properties [34]–[36]. Each immunogen was tested as a monomer or a trimer, and with two different trimer plus adjuvant regimens in New Zealand White rabbits. All four of the gp140 immunogens in both forms were immunogenic, with immunizations resulting in high titers of binding antibodies. The magnitude of the binding antibody responses elicited by monomers and trimers were statistically similar, as were the binding titers of antibodies elicited by the full adjuvant (4A) versus half adjuvant (2A) protocol. Similarly, the neutralizing antibody responses that were elicited by monomers versus trimers and half adjuvant versus full adjuvant were similar.

Subject VC10042 entered observation at approximately 19 years post infection with HIV-1 [34], and the Envs used in this study were isolated from subject VC10042 after more than 22 years after the subject acquired HIV-1 [35]. Thus, it is probable that these are not the Envs that drove the initial development of neutralizing breadth in this subject. However, three of the four Envs (excluding 10042.05) are sensitive to autologous neutralizationfrom the contemporaneous plasma, and two of the Envs, 10042.e1a and 10042.08, are very sensitive to neutralization by anti-CD4-BS monoclonal antibodies [35]. In the case of neutralization-resistant *env* 10042.05, introducing the R373M mutation (which is known to alter exposure of the CD4 binding pocket [54]) to create 10042.05.RM makes it exquisitely sensitive to the contemporaneous plasma and moderately sensitive to anti-CD4-BS MAbs [35]. Thus, at least three of the four Envs express the epitopes necessary to mediate potent, broadly neutralizating activity through the CD4-BS.

The VC10042-derived Envs evolved over a long period of time in the presence of strong anti-CD4-BS response and reflect a historical accumulation of escape mutations. Such escape likely involved increasing glycosylation, mutating the variable loops, and masking and/or mutating narrow neutralizing epitopes. Our goal was to evaluate whether such Envs developed unique antigenic properties through these processes that could be leveraged toward focusing the immune response on conserved epitopes and could be used to elicit broad neutralizing responses. Thus, our approach in testing the ‘end result’ of co-evolution in the context of anti-CD4-BS serum bNAb responses differs significantly from the approach in which temporally-spaced Envs isolated from broad neutralizers may be used to drive the stimulation and maturation of neutralizing breadth (including specific B cell lineages) by vaccination [55].

In addition, our study has several key differences from the previous studies involving the gp140_R2_ immunogen [32], [33], [56]. The parental *env* clones from VC10042 used in this study are all known to be CD4-dependent, CCR5-tropic viruses [35], whereas gp140_R2_ was derived from a CD4-independent *envelope* [57]. Also, we have extensively characterized the cross-clade broadly neutralizing activity in subject VC10042 and have mapped the activity to the CD4-BS [34], whereas it is not clear whether the gp140_R2_ donor plasma (HNS2) has been screened against modern tiered neutralization panels or whether the neutralizing activity has been mapped to a specific epitope [32], [33], [56], [58], [59]. Lastly, although the gp140_R2_ and VC10042-derived gp140s are similar in amino acid length, the VC10042 Envs are more heavily glycosylated and have 2 additional (10042.08) or 5 additional (10042.05 and 10042.05.RM) NLGS sites compared to gp140_R2_. Thus, phenotypically gp140_R2_ and the VC10042-derived gp140s appear to be quite different. Despite these differences, it is clear that both gp140_R2_ and the VC10042 Envs appear to contain unique (albeit different) antigenic characteristics that make them well-suited to elicit cross-reactive antibodies by vaccination.

It is interesting that we were not able to identify an advantage to providing adjuvant with all four immunizations, rather than just with the first two immunizations. One explanation for this observation may be that adjuvant is not critical after the boost immunization and anamnestic response. However, the readout used to determine this (serum binding titers and neutralization activity) may not provide the definitive answer. It is possible that there are differences between the circulating BCR repertoires in the two immunization groups that may be reflected in the level of somatic hypermutation that the vaccinations achieved or in the specific pathways of BCR evolution. Future efforts in this respect are focused on evaluating vaccine regimens not only by neutralization potential that can be recorded using *in-vitro* assays, but also by evaluating the evolution of the circulating anti-immunogen BCR repertoire over time at the IgH/K/L sequence level.

In many cases the serum neutralizing activity did not reach 50%, indicating that although there is a high degree of breadth in several of the immunization groups, the responses are of low potency. However, the fact that we observe neutralizing activity against a wide array of heterologous isolates suggests the presence of antibodies in the immune serum that target conserved epitopes. There are several potential explanations for why these animals exhibit a high degree of breadth but low potency, including that 1) neutralizing antibody responses to conserved epitopes are developing in these animals, but they are not mature enough to display high binding avidity and neutralizing potency; and 2) that broadly neutralizing antibodies are generated but are present at sub-effective concentrations in the serum. Future efforts will be focused on determining which of these possibilities occurred.

In addition to neutralizing antibodies, all four of the VC10042-derived gp140 Envs elicited cross-reactive binding antibodies that recognize an epitope in the V1V2, as presented on the MLV gp70-V1V2 scaffold. These antibodies are potentially similar to antibodies that were recently reported to be associated with a decreased risk of HIV-1 acquisition in the RV144 trial, and are considered to be a desirable component of HIV-1 vaccine candidates moving forward [3]. The highest anti-V1V2 titers were elicited by VC10042.05 and VC10042.05.RM gp140s, which were also the same gp140s that elicited the broadest NAb responses in rabbits. Thus, if VC10042.05 and VC10042.05.RM gp140s can be modified to increase their ability to elicit more potent bNAb responses without diminishing their ability to elicit anti-V1V2 antibodies, they would be promising immunogens that would warrant more extensive pre-clinical evaluation. The development of Env vaccine immunogens that could reliably elicit bNAbs and anti-V1V2 responses would represent a significant advancement for HIV-1 vaccine development.

## Supporting Information

File S1 **Tables S1, S2 and Figures S1–S7.** Table S1. Amino acid sequences of the linear peptides used in Luminex binding assays. Table S2. ELISA endpoint titers following immunization with VC10042-derived Envs. Figure S1. Sequences of the MLV-gp70-V1V2 protein scaffolds used in this study. The tPA leader sequence, which is cleaved off during protein maturation, is shown in bold type. The portion corresponding to the HIV-1 V1V2 region is underlined. Figure S2. Immunization schedule for 2 adjuvant (2A) regimen. Figure S3. Immunization schedule for 4 adjuvant (4A) regimen. Figure S4. Antigenic profile of trimeric and monomeric 10042.05 gp140. Binding of well-characterized monoclonal anti-Env antibodies was measured by ELISA. (t) = trimeric gp140; (m) = monomeric gp140. Figure S5. Antigenic profile of trimeric and monomeric 10042.05.RM gp140. Binding of well-characterized monoclonal anti-Env antibodies was measured by ELISA. (t) = trimeric gp140; (m) = monomeric gp140. Figure S6. Antigenic profile of trimeric and monomeric 10042.08 gp140. Binding of well-characterized monoclonal anti-Env antibodies was measured by ELISA. (t) = trimeric gp140; (m) = monomeric gp140. Figure S7. Antigenic profile of trimeric and monomeric 10042.e1a gp140. Binding of well-characterized monoclonal anti-Env antibodies was measured by ELISA. (t) = trimeric gp140; (m) = monomeric gp140.
(DOCX)
