# Supplementary material for: Soluble HIV-1 Envelope Immunogens Derived from an Elite Neutralizer Elicit Cross-Reactive V1V2 Antibodies and Low Potency Neutralizing Antibodies
Source: PLoS One. 2014 Jan 23;9(1):e86905. doi: 10.1371/journal.pone.0086905 (PMC3900663; doi:10.1371/journal.pone.0086905)
Supplement: File S1 — Tables S1, S2 and Figures S1–S7. Table S1. Amino acid sequences of the linear peptides used in Luminex binding assays. Table S2. ELISA endpoint titers following immunization with VC10042-derived Envs. Figure S1. Sequences of the MLV-gp70-V1V2 protein scaffolds used in this study. The tPA leader sequence, which is cleaved off during protein maturation, is shown in bold type. The portion corresponding to the HIV-1 V1V2 region is underlined. Figure S2. Immunization schedule for 2 adjuvant (2A) regimen. Figure S3. Immunization schedule for 4 adjuvant (4A) regimen. Figure S4. Antigenic profile of trimeric and monomeric 10042.05 gp140. Binding of well-characterized monoclonal anti-Env antibodies was measured by ELISA. (t) = trimeric gp140; (m) = monomeric gp140. Figure S5. Antigenic profile of trimeric and monomeric 10042.05.RM gp140. Binding of well-characterized monoclonal anti-Env antibodies was measured by ELISA. (t) = trimeric gp140; (m) = monomeric gp140. Figure S6. Antigenic profile of trimeric and monomeric 10042.08 gp140. Binding of well-characterized monoclonal anti-Env antibodies was measured by ELISA. (t) = trimeric gp140; (m) = monomeric gp140. Figure S7. Antigenic profile of trimeric and monomeric 10042.e1a gp140. Binding of well-characterized monoclonal anti-Env antibodies was measured by ELISA. (t) = trimeric gp140; (m) = monomeric gp140. (DOCX) [file pone.0086905.s001.docx]

Table S1. Amino acid sequences of the linear peptides used in Luminex binding assays.

Table S2. ELISA endpoint titers following immunization with VC10042-derived Envs.

| **Immunogen** | | **VC10042.05.RM (t)^1^ 2A^2^** | | | **VC10042.05 (t) 2A** | | | **VC10042.05.RM (m)^3^ 2A** | | | **VC10042.05 (m) 2A** | | |
| --- | --- | --- | --- | --- | --- | --- | --- | --- | --- | --- | --- | --- | --- |
| **TITER** | Animal | 27088 | 27089 | 27090 | 27091 | 27092 | 27093 | 27216 | 27217 | 27218 | 27219 | 27220 | 27221 |
|  | Post 2nd | 5.67 | 4.92 | 5.37 | 5.57 | 5.42 | 5.07 | 5.26 | 5.26 | 4.95 | 4.61 | 4.73 | 4.73 |
|  | Post 3rd | 5.69 | 5.41 | 5.63 | 5.55 | 5.15 | 5.01 | 5.64 | 5.39 | 5.14 | 5.46 | 5.40 | 5.63 |
|  | Post 4th | 5.08 | 5.00 | 4.96 | 4.67 | 5.12 | 4.93 | 5.53 | 5.64 | 5.43 | 5.38 | 5.38 | 4.66 |
|  | | | | | | | | | | | | | |
| **Immunogen** | | **VC10042.08 (t) 2A** | | | **VC10042.e1a (t) 2A** | | | **VC10042.08 (m) 2A** | | | **VC10042.e1a (m) 2A** | | |
| **TITER** | Animal | 28249 | 28250 | 28251 | 28252 | 28253 | 28254 | 28255 | 28256 | 28257 | 28258 | 28259 | 28260 |
|  | Post 2nd | 5.34 | 5.51 | 5.30 | 5.24 | 4.91 | 5.35 | 4.74 | 5.11 | 4.96 | 4.33 | 4.75 | 4.97 |
|  | Post 3rd | 5.43 | 5.45 | 5.23 | 5.11 | 5.00 | 5.32 | 4.63 | 4.84 | 5.31 | 4.21 | 3.99 | 4.33 |
|  | Post 4th | 5.64 | 5.16 | 4.89 | 5.21 | 5.03 | 5.47 | 5.09 | 4.90 | 4.60 | 5.09 | 4.79 | 4.47 |
|  | | | | | | | | | | | | | |
| **Immunogen** | | **VC10042.08 (t) 4A^4^** | | | **VC10042.e1a (t) 4A** | | | **VC10042.05 (t) 4A** | | | **VC10042.05.RM (t) 4A** | | |
| **TITER** | Animal | 28261 | 28262 | 28263 | 28264 | 28265 | 28266 | 28267 | 28268 | 28269 | 28270 | 28271 | 28272 |
|  | Post 2nd | 5.13 | 5.29 | 4.91 | 4.54 | 5.21 | 5.44 | *Deceased* | 5.51 | 5.39 | 5.60 | 5.30 | 4.26 |
|  | Post 3rd | 5.16 | 5.02 | 5.02 | 4.64 | 5.03 | 5.03 |  | 5.14 | 5.65 | 5.61 | 5.44 | 5.30 |
|  | Post 4th | 5.65 | 5.2 | 4.92 | 4.83 | 5.08 | 5.08 |  | 4.83 | 5.20 | 5.57 | 5.57 | 5.02 |

^1^(t) = trimeric gp140

^2^(2A) = adjuvant at only the first two immunizations

^3^(m) = monomeric gp140

^4^(4A) = adjuvant at all four immunizations

**Carbonetti, et al., Figure S1**

MLV-gp70-V1V2 Consensus Clade A

**MDAMKRGLCCVLLLCGAVFVSPSAS**AAPGSSPHHVYNITWEVTNGDRETVWAISGNHPLWTWWPVLTPDLCMLALSGPPHWGLEYQAPYSSPPGPPCCSGSSGSSAGCSRDCDEPLTSLTPRCNTAWNRLKLDQVTHKSSEGFYVCPGSHRPREAKSCGGPDSFYCASWGCETTGRVYWKPSSSWDYITVDNNLTTSQAVQVCKDNKWCNPLAIQFTNAGKQVTSWTTGHYWGLRLYVSGRDPGLTFGIRLRYQNLGPRVPIGPNPVLADQLSLPRPNPLPKPAKSPPASVKLTPLCVTLNCSNANTTNNSTMEEIKNCSYNITTELRDKTQKVYSLFYKLDVVQLDESNKSEYYYRLINCNTSAITQA

MLV-gp70-V1V2 Consensus Clade B

**MDAMKRGLCCVLLLCGAVFVSPSAS**AAPGSSPHQVYNITWEVTNGDRETVWAISGNHPLWTWWPVLTPDLCMLALSGPPHWGLEYQAPYSSPPGPPCCSGSSGNVACARDCNEPLTSLTPRCNTAWNRLKLDQVTHKSSEGFYVCPGSHRPREAKSCGGPDSFYCASWGCETTGRVYWKPSSSWDYITVDNNLTSNQAVQVCKDNKWCNPLAIRFTNAGKQVTSWTTGHYWGLRLYVSGQDPGLTFGIRLSYQNLGPRIPIGPNPVLADQLSFPLPNPLPKPAKSPPASLKPCVKLTPLCVTLNCTDLMNATNTNTTIIYRWRGEIKNCSFNITTSIRDKVQKEYALFYKLDVVPIDNDNTSYRLISCNTSVITQACPKVS

MLV-gp70-V1V2 Consensus Clade C

**MDAMKRGLCCVLLLCGAVFVSPSAS**AAPGSSPHQVYNITWEVTNGDRETVWAISGNHPLWTWWPVLTPDLCMLALSGPPHWGLEYQAPYSSPPGPPCCSGSSGNVAGCARDCNEPLTSLTPRCNTAWNRLKLDQVTHKSSEGFYVCPGSHRPREAKSCGGPDSFYCASWGCETTGRVYWKPSSSWDYITVDNNLTSNQAVQVCKDNKWCNPLAIRFTNAGKQVTSWTTGHYWGLRLYVSGQDPGLTFGIRLSYQNLGPRIPIGPNPVLADQLSFPLPNPLPKPAKSPPASLKPCVKLTPLCTNVTNTTVNNNSTNMTGEMKNCSFNITTEIRDKKQKEYALFYKLDIVPLNNSSEYRLINCNTSAITQACPKVS

Carbonetti, et al., Figure S2

Carbonetti, et al., Figure S3

Carbonetti, et al., Figure S4

Carbonetti, et al., Figure S5

Carbonetti, et al., Figure S6.

Carbonetti, et al., Figure S7
